# Design and Molecular Modeling of Abiraterone-Functionalized Gold Nanoparticles

**DOI:** 10.3390/nano8090641

**Published:** 2018-08-22

**Authors:** Elżbieta U. Stolarczyk, Marta Łaszcz, Andrzej Leś, Marek Kubiszewski, Krzysztof Kuziak, Katarzyna Sidoryk, Krzysztof Stolarczyk

**Affiliations:** 1 R&D Analytical Chemistry Department, Pharmaceutical Research Institute, 8 Rydygiera Street, 01-793 Warsaw, Poland; m.laszcz@ifarm.eu (M.Ł.); a.les@ifarm.eu (A.L.); m.kubiszewski@ifarm.eu (M.K.); k.kuziak@ifarm.eu (K.K.); 2 Faculty of Chemistry, University of Warsaw, 1 Pasteura Street, 02-093 Warsaw, Poland; kstolar@chem.uw.edu.pl; 3 Chemistry Department, Pharmaceutical Research Institute, 8 Rydygiera Street, 01-793 Warsaw, Poland; k.sidoryk@ifarm.eu

**Keywords:** AuNPs-gold nanoparticles, AB-abiraterone, conjugates, NP-based system, thermogravimetry, Raman spectroscopy, powder diffraction, transmission electron microscopy, density functional theory

## Abstract

The aim of our work was the synthesis and physicochemical characterization of a unique conjugate consisting of gold nanoparticles (AuNPs) and a pharmacologically active anticancer substance abiraterone (AB). The direct coupling of AB with gold constitutes an essential feature of the unique AuNPs–AB conjugate that creates a promising platform for applications in nanomedicine. In this work, we present a multidisciplinary, basic study of the obtained AuNPs–AB conjugate. Theoretical modeling based on the density functional theory (DFT) predicted that the Au_n_ clusters would interact with abiraterone preferably at the N-side. A sharp, intense band at 1028 cm^−1^ was observed in the Raman spectra of the nanoparticles. The shift of this band in comparison to AB itself agrees well with the theoretical model. AB in the nanoparticles was identified by means of electrochemistry and NMR spectroscopy. The sizes of the Au crystallites measured by XRPD were about 9 and 17 nm for the nanoparticles obtained in pH 7.4 and 3.6, respectively. The size of the particles as measured by TEM was 24 and 30 nm for the nanoparticles obtained in pH 7.4 and pH 3.6, respectively. The DLS measurements revealed stable, negatively charged nanoparticles.

## 1. Introduction

**Figure 1:**
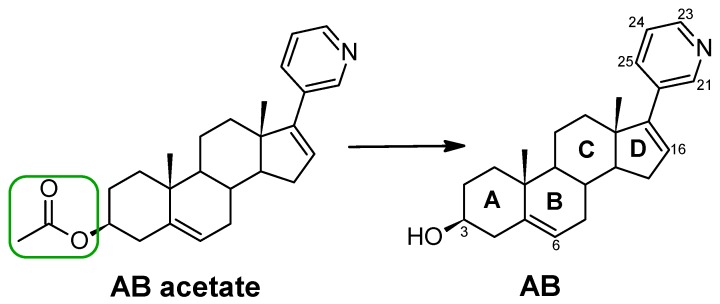
Structures of abiraterone acetate (AB acetate) and abiraterone (AB). Four rings of the androsta-5,16-dien-3β-ol moiety are denoted as A, B, C, and D.

Abiraterone (AB) is administered as an acetate ester prodrug which is rapidly converted in vivo to abiraterone [1]. Abiraterone is an irreversible inhibitor of 17α-hydroxylase/C17,20-lyase (CYP17), a key enzyme in the production of androgens in the testes and adrenal glands. The structural formula of abiraterone acetate and its active metabolite, abiraterone, is presented in Figure 1.

Challenges related to modern drug form technology consist, among other things, in using techniques and technologies that allow us to deliver the drug directly to the drug target, extend the time of the API’s (Active Pharmaceutical Ingredient) activity in the drug target, and influence the API’s distribution. In the light of the above, nanotechnology gains primary importance [2,3,4,5]. Inorganic nanoparticles and their combinations with organic substances in the hybrid form are characterized by unique physical, chemical, and biological properties. A shift into the nano-scale causes a significant increase in the specific grain boundary area per unit volume. This in turn results in a remarkable increase of the reactivity of the material, adsorption, anti-microbiological, and anticancer activity [6,7,8,9,10]. Nanoparticles improve therapy efficacy by transporting the drug to the drug target, thus increasing treatment safety. By stabilizing the drug on the nanoparticle surface, the solubility of the conjugates is improved. A bigger surface-to-volume ratio for nanomaterials means that the number of drug particles bound to the nanoparticles increases. That is why efforts are made to design new drug delivery systems, especially for anticancer drugs, such as abiraterone, which are poorly soluble and have multiple side effects.

Unfortunately, their synthesis, desirable modification, and characterization remain difficult. A particular synthetic route has to be carefully selected because it has a direct influence on the shape and size of the nanoparticles and their stability and presence and the orientation of the active substance that stabilizes the area of the nanoparticle and influences the properties of the unique nanoparticle [8]. In most cases, the mechanism of the interactions between gold nanoparticles (AuNPs) and active substances remains a mystery, although it could be useful for understanding the release mechanism of the active substance or its activity within cells. The interaction between gold clusters, surfaces, or AuNPs and biomolecules has attracted the attention of scientists for many years: [11] (Au_8_, Au_20_ with alanine, tryptophan), [12] (gold surface with cytosine, 5-methylcytosine), [13] (Au_13_ with guanine), [14] (Au_n_ with guanine-cytosine, adenine-uracil, n = 5–10), [15] (Au_1_ with pyridine). The first experimental study that showed a direct interaction of pyridine with the gold surface was published in 1991 [16].

Our article reports an original synthesis of a new kind of material composed of gold nanoparticles conjugated with anticancer abiraterone drugs. The aims of our work were as follows: to obtain gold nanoparticles as potential carriers of the pharmacologically active substance abiraterone, and to study interactions in the Au_n_–abiraterone and Au_n_–abiraterone acetate conjugates. The nanomaterials are characterized by various techniques with the support of theoretical calculations. We have applied the density functional theory (DFT), which is a quantum-mechanical method offering tools for the investigation of interatomic and intermolecular interactions in new molecular systems. The studies employing DFT enabled us to provide reliable three-dimensional (3-D) molecular structures and numerous spectral properties of biologically and pharmacologically active molecules as was shown in the literature [11,12,13,14,15,16]. Bearing this in mind, we hope to obtain by means of DFT a reasonable estimation of the molecular structure, binding energy, and vibrational spectra of the Au_n_–AB conjugates. We also present physicochemical characteristics of the conjugates. In this work, we have identified the AuNPs–AB conjugates by electrochemical, NMR, UV-Vis, and XRPD measurements. The obtained conjugates have been characterized by Raman, DLS, zeta-potential, and TGA techniques. Multi-disciplinary studies on new NP-based systems are particularly important before a new product of pharmaceutical quality is developed. The presented research allows us to gain knowledge on the technology of the abiraterone–AuNPs conjugates. To the best of our knowledge, no syntheses or physicochemical characterization of AuNPs–AB conjugates have been published in the literature to date.

## 2. Materials and Methods

### 2.1. Materials

Tetrachloroauric acid (HAuCl_4_) and DMSO-*d*_6_ were purchased from Sigma Aldrich (Saint Louis, MO, USA). Abiraterone and acetate abiraterone (batch Nos. 219595 and 217728, respectively) were obtained from Molekula SRL (Rimini, Italy). Dimethyl sulfoxide and dimethylformamide (GC-headspace tested 99.9%) were purchased from Aldrich. Other chemical reagents used in this study were obtained from POCH (Gliwice, Poland). All solutions were prepared with ultrapure water (18.2 MΩ) purified on the Polwater System D-300 (Cracow, Poland). Deuterated solvents for NMR spectroscopy were purchased from ARMAR AG (Döttingen, Switzerland).

### 2.2. UV-Vis Spectroscopy

UV-Vis spectroscopic measurements were obtained by using a UV-Vis spectrophotometer, Evolution 220 series (Thermo Scientific, Waltham, MA, USA), with a 1-cm quartz cell in the wavelength range from 220 to 750 nm.

### 2.3. Nuclear Magnetic Resonance (NMR) Spectroscopy

The ^1^H NMR spectra of the studied AuNPs–AB and AuNPs–AB acetate samples were performed using Varian Gemini-2000 and Bruker Avance 500 (Bruker Corporation, Billerica, MA, USA) spectrometers with the resonance frequency of 200 and 500 MHz, respectively. The sample was dissolved in deuterated DMSO-*d*_6_ with a small addition of D_2_O where the residual signal of the solvent DMSO-*d*_6_ was simultaneously the internal standard of the chemical shift (2.49 ppm). Standard measurement parameters were used.

### 2.4. Raman Spectroscopy

The studied solution of the nanoparticle was placed in a metal cup and dried in a drying chamber at 80 °C.

The FT Raman spectra were recorded on a Nicolet NXR 9650 instrument (Thermo Scientific, Waltham, MA, USA) using 1064 nm excitation from the Nd:YVO4 laser in the range from 3700 to 150 cm^−1^ with the spectral resolution of 4 cm^−1^. For the nanoparticle spectrum, 1000 scans were recorded with the laser power of 0.1 W. For the AB and AB acetate spectra, from 100 to 300 scans were recorded with the laser power from 0.4 to 0.8 W.

### 2.5. Transmission Electron Microscopy (TEM)

TEM photos were taken shortly after the synthesis of nanoparticles. The TEM measurements were made using a Carl Zeiss Libra microscope. The accelerating voltage was 120 kV. A small volume of the resulting nanoparticles solution was diluted with 1-butanol, and then two drops were placed on a copper TEM 400 mesh coated with carbon. The sample was protected from external factors and allowed to dry under air.

### 2.6. Dynamic Light Scattering (DLS) and Electrophoretic Light Scattering (ELS)

Dynamic light scattering (DLS) and electrophoretic light scattering (ELS) measurements of the synthesized gold nanoparticles were carried out using a Zetasizer Nano ZS (Malvern Instruments Ltd., Malvern, UK). The angle of the incident light was 173°. The mean size and zeta potential were calculated for each specimen that underwent five measurements.

### 2.7. Electrochemical Measurements

Electrochemical measurements were performed on the ECO Chemie Autolab potentiostat (Utrecht, The Netherlands) at the temperature of 22 ± 2 °C using chronoamperometry in a three-electrode system with the electrodes: working: glassy carbon disk electrode (GCE, BASi); reference: silver-chloride electrode, Ag/AgCl (saturated KCl); and counter: a platinum foil. Electrochemical measurements were recorded in the anaerobic environment obtained by passing argon through the DMSO solution with 0.1 M tetrabutylammonium hexafluorophosphate (TBAHFP) for about 20 min. All current densities were calculated using the geometric electrode area (0.071 cm^2^) of the GCE working electrode.

### 2.8. Thermogravimetry (TGA) Analysis

The TGA measurements were performed by means of a TGA/SDTA851 cell (MettlerToledo GmbH, Schwerzenbach, Switzerland). About 1 mg of the samples was weighed into aluminum crucibles (40 µL). The samples were heated from 30 to 600 °C at 10 °C/min in the nitrogen atmosphere (60 mL/min). The measurements were blank curve corrected.

### 2.9. Powder X-ray Diffraction (XRPD)

The diffractograms were recorded on the MiniFlex diffractometer (Rigaku Coporation, Tokyo, Japan) using CuKα1 radiation. The nanoparticles as well as the mixtures were loaded on glass sample holders and their surfaces were smoothed by glass plates. The instrument was operated in the range from 3° to 90° with the scanning speed of 0.5°/min and the step angle of 0.02°. The FWHM (full width at half maximum) value for the Au(111) peak was calculated by fitting the split Pearson VII function to the peak shape. The average size of the Au crystallites was estimated from the Scherrer formula assuming that the K constant is 0.94. The following phase identification was performed by means of the PDF-2 Database [17]: Au (PDF No. 04-0784), NaCl (PDF No. 05-0628). Abiraterone acetate was identified by comparison with a simulated diffractogram obtained on the basis of X-ray single crystal studies. These data are available from CCDC (Cambridge Crystallographic Data Centre, Cambridge, UK), No. 1484555 [18].

### 2.10. Theoretical Calculations

The 3-D geometry, harmonic frequencies, and Raman activities were predicted using the Density Functional Theory and the B3LYP functional with the 6-31G(d,p) Gaussian basis sets for the H, C, N, O atoms and the LANL2DZ effective core potential for the Au atoms. The geometry optimization used the algorithm of Berny implemented in the Gaussian G09 suite of the programs [19]. The optimal geometry was characterized with positive frequencies. The following problems have been considered:

- Determination of the molecular structure of small Au_n_ clusters (n = 5, 13, 20) interacting with abiraterone and abiraterone acetate molecules (or their reduced models). Such systems are models of the hypothetical gold nanoparticles (AuNPs) conjugates with abiraterone in the human body.
- As the 3-D geometry of the abiraterone molecule recalls a roller-like structure, it is interesting to determine whether abiraterone or abiraterone acetate molecules bind the Au_n_ cluster via N-terminal nitrogen or via the OH or C=O-terminal groups. The orientation of abiraterone with respect to the gold cluster is expected to be an essential feature determining its consecutive interaction with the components of living cells.
- The molecular structure of the AuNPs–abiraterone conjugates can be investigated with Raman spectroscopy. One can model Raman spectra with the use of the theoretically predicted band frequencies and intensities of small Au_n_ clusters interacting with the abiraterone molecule. The theoretically predicted normal modes in the spectral region 1000–1800 cm^−1^ can help assign most of the bands in the experimental Raman spectrum and draw conclusions regarding the possible mode of gold–abiraterone interactions. The Raman intensities were obtained following the formula derived from the intensity theory of Raman scattering [20,21] using the laser beam excitation energy of 1064 nm. The intense band assignment was supported by the visual inspection of the normal modes with the use of the Jmol software [22].

### 2.11. Synthesis of Gold Nanoparticles with Abiraterone (AuNPs–AB)

Briefly, all glassware was cleaned in aqua regia (three parts HCl, one part HNO_3_), rinsed with H_2_O, next with acetone, and then dried prior to use. This study uses a chemical method for obtaining AuNPs based on the Au^3+^ ions reduction to Au^0^. The synthesis of the AuNPs–AB conjugates was carried out in distilled water (mixing the ingredients gives a pH 3.6) and in pH 7.4 (with the addition of NaOH). The other steps of the synthesis were the same. The AuNPs–AB conjugates were prepared as follows: AB (1.0% m/v, 5 mL DMF) was added with stirring to the HAuCl_4_∙3H_2_O solution (10 mg, 90 mL) and heated to 100 °C. Next, 5 mL of DMSO was added. While stirring the reaction mixture, the color of the boiling solution changed from yellow through purple to wine-purple. The solution was boiled for 30 min and cooled down to room temperature. Subsequently, the solution was lyophilized. Then, 50 mL of *n*-BuOH was added to the lyophilized mixture. The solution was centrifuged twice at 14,000 rcf for 60 min and the precipitate was rinsed with *n*-BuOH between centrifugations. Next, the precipitate of AuNPs–AB was separated and dried. The mixture was centrifuged to separate the unadsorbed AB from the AuNPs–AB conjugates. The efficiency of the purification of the precipitate from free abiraterone was controlled by a UV-Vis method to obtain supernantants without free AB. UV-Vis, Raman, NMR, electrochemistry, XRPD, and TGA measurements of abiraterone bound to the AuNPs were recorded. The surface charge of the AuNPs after abiraterone loading was determined by measuring the zeta potential. The particle size of AuNPs–AB was determined by DLS and TEM.

## 3. Results and Discussion

This paper presents a novel approach consisting in conjugating abiraterone (AB) with gold nanoparticles (AuNPs), which is expected to have plausible tumor-targeting ability and to improve the efficacy of cancer therapy.

### 3.1. Synthesis Development

Initial research focused on the choice of an adequate substance for the nanoparticle synthesis. Two compounds, i.e., abiraterone acetate and abiraterone, Figure 1, were selected for the synthesis of the conjugates. The synthesis of nanoparticles containing abiraterone acetate required a minimum of 1 h of the reaction time, and slight colouring of the solution suggesting the presence of the nanoparticles was observed after 1.5 h. Furthermore, when abiraterone acetate was used, the yield of gold nanoparticles was low and only a small amount of the desired product was obtained. From this observation, it has been suggested that a hydroxyl group (OH) plays a significant role during the reaction. The results for the AuNPs–AB acetate conjugate are presented in the Supplementary Material (SM). Further studies were carried out involving the synthesis of the nanoparticle containing abiraterone (AB).

**Figure 2:**
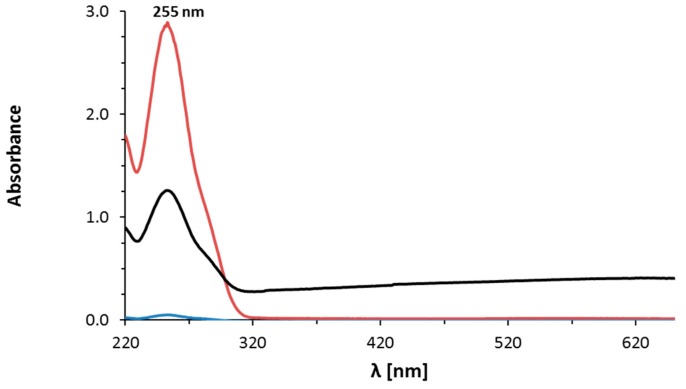
Control of abiraterone in the supernatant by the UV-Vis technique: after one centrifugation (red line); after two centrifugations (black line); after three centrifugations (blue line): below the limit of detection (LOD).

Abiraterone is hydrophobic; therefore, it was important to select an additional solvent that could dissolve the test compound in the aqueous reaction. Widely used DMSO, DMF, and THF solvents have been tested. The best results were achieved when DMF and DMSO were used. It was found that AB was most easily dissolved in DMF, and the addition of DMSO to the solution increased the reaction speed. Nanoparticles formed faster and acquired a more intense raspberry colour when DMSO was added. During the optimization of the reaction, it was observed that the order of adding DMSO was of great significance. When DMSO was added first to the HAuCl_4_∙3H_2_O solution, followed by the AB solution in DMF, a brown mixture was obtained because of the formation of complex Au–DMSO. Therefore, it was important to add the AB solution in DMF to the HAuCl_4_∙3H_2_O solution then DMSO. The AuNPs–AB conjugates can be created using DMSO alone, as well as DMF alone, too. The next step of the investigation was to purify the obtained crude nanoparticiples from the excess amount of AB. Several known solvents were tested, such as DMSO, EtOH, THF, DMF, *n*-BuOH, and MeCN. *N*-BuOH proved to be the best solvent as regards removing the non-AuNPs–AB components; moreover, when this solvent was used, the yield of the purification process was the highest. Briefly, the reaction mixture was lyophilized, next the crude material was treated with *n*-BuOH and centrifuged. An optimal centrifugation process was obtained based on the tests of different centrifugation speeds and different duration times of the process. The nanoparticles were isolated by centrifuge set at the maximum speed of 14,000 rcf and the duration time of 60 min. This purification process was repeated three times and controlled in the supernatant by the UV-Vis technique (Figure 2). Elements of the validation of the UV-Vis method for the determination of AB in the supernatant are presented in the Supplementary Material (Tables S1–S3, Figure S1). It was proved that the method was linear, sensitive, and had no systematic errors in the range of 4–50 µg/mL. The limits of detection and quantitation were 3 µg/mL and 4 µg/mL, respectively.

**Figure 3:**
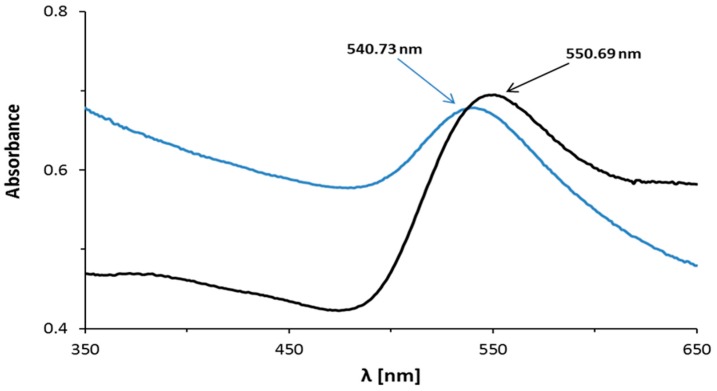
UV-Vis spectra of the AuNPs–AB conjugate in pH 3.6: the blue line denotes reaction mixtures; the black line denotes nanoparticle precipitate.

**Figure 4:**
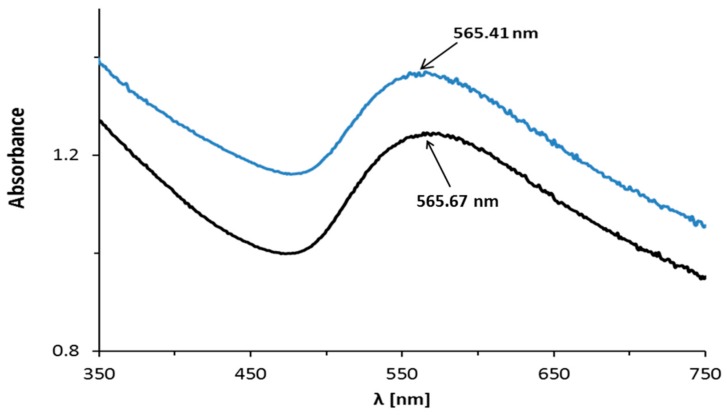
UV-Vis spectra of the AuNPs–AB conjugate in pH 7.4: the blue line denotes reaction mixtures; the black line denotes nanoparticle precipitate.

The absorption maxima were observed on the UV-Vis spectra of the AuNPs–AB pH 3.6 and AuNPs–AB pH 7.4 reaction mixtures at 541 and 565 nm, respectively (Figure 3 and Figure 4, blue lines). These bands are derived from the formed nanoparticles.

In comparison to the nanoparticles in the reaction mixture at pH 3.6, only small changes in the UV-Vis maximum absorption band of the nanoparticle precipitate were observed, so the aggregation was small during the purification and centrifugation of this solution (Figure 3). The aggregation was not observed during the purification and centrifugation of the reaction mixture in pH 7.4 (Figure 4).

At pH 7.4, upon abiraterone binding to the gold nanoparticles’ surface, the localized surface plasmon resonance (LSPR) spectrum was red-shifted by a few nanometers compared to pH 3.6. Moreover, the band at pH 7.4 was wider. This shift is a result of an increase in the local refractive index at the gold nanoparticle surface [23]. It suggests a larger nanoparticle size. A similar dependency was observed for the DLS technique (Table 1). The DLS technique measures the hydrodynamic size of the nanoparticle core together with the coating.

**Figure 5:**
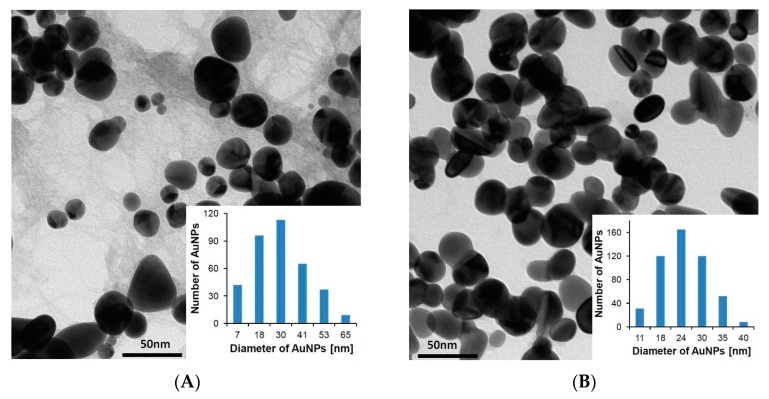
TEM images and histograms of (**A**) AuNPs–AB pH 3.6 and (**B**) AuNPs–AB pH 7.4 particle size distribution.

Other differences were observed in the Raman spectra (Section 3.4), XRPD analysis (Section 3.3), and TEM figures. TEM was used to determine the size and structural morphology of the AuNPs–AB nanoparticles (Figure 5). Black, ball-shaped objects appeared in the pictures, which corresponded to the cores of the gold nanoparticles. Figure 5 also shows histograms that were prepared after measuring 400 diameters of the gold nanoparticles. For AuNPs–AB pH 3.6, the particle diameters belong to the 7–65 nm interval with the maximum at 30 nm. The corresponding values at pH 7.4 are 11–40 nm, with the maximum at 24 nm. The nanoparticles are roughly monodispersed. However, in the case of AuNPs–AB at pH 3.6, the dispersion is larger. A similar relation occurs for the maximum particle diameter. Particle sizes measured with TEM are much smaller than in the case of DLS, which is due to the loss of the hydration coating of the nanoparticles during TEM measurements and the lack of mutual interactions of the nanoparticles present in the solvent. When nanoparticles adsorb liquids intrinsically, the diameter changes a lot. Nanoparticles in water can be referred to as clouds. In our opinion, the increase of the nanoparticle size which has been observed with the DSL measurements is not an aggregation, but most likely the result of creating the hydrodynamic diameter (responsible for the diffusion in liquids) [24]. XRPD was used to calculate the average size of the Au crystallites. A comparison of the AuNPs–AB precipitates obtained for the two different pH values suggests a higher stability of the pH 7.4 conjugate as well as a smaller nanoparticle size and a smaller Au crystallite size. These differences are probably related to the local intermolecular hydrogen bond of the O–H–N type that occurs in pH 7.4. The protonation of the pyridine group present in the abiraterone molecule depended on the pH of the reaction mixture. In pH 3.6, which is a natural pH obtained after mixing the reaction ingredients, protonation of the nitrogen atom in the pyridine ring occurs. In pH 7.4, the nitrogen atom appears in a neutral form suitable for hydrogen bond formation. This can be reflected as a bigger nanoparticle size measured by means of the DLS and UV-Vis techniques.

### 3.2. Theoretical Studies

The intermolecular interaction energy, E_int_, was estimated using the counterpoise correction method (to reduce the basis set superposition error) following the formula: E_int_ = E(Au_n_-AB) − E(Au_n_) − E(AB), where all the energies were calculated using the same Gaussian basis set of the conjugate.

Two geometries of the Au_n_–AB conjugates were considered: the structure where the Au_n_ cluster is approached from the N-nitrogen side of the abiraterone molecule (denoted as (N)AB), and another structure where the Au_n_ cluster is approached at the OH-group or the C=O group side of abiraterone or abiraterone acetate molecules (denoted as (OH)AB or (O=C)AB). The two geometries are visualized in Table S4 in the Supplementary Material. The calculations were performed for the Au_5_–abiraterone and Au_13_–abiraterone conjugates and for the Au_5_–abiraterone acetate conjugates. In order to assess the Au_20_–abiraterone interaction energy, a reduced model was used due to an excessive size of the system for the present theoretical methodology. The abiraterone molecule was replaced by the pyridine molecule in order to simulate abiraterone interaction via the N-side. The abiraterone interaction via the OH-group side was simulated with the interaction of the 3-cyclohexen-1-ol at the OH-side. A comparison of the relative energies of the conjugates as well as selected intermolecular interaction energies are presented in Table 2, while the details are given in Table S5 in the Supplementary Material.

The interaction energies, also called the binding energies (BE), fall into an interval of about 20 ± 5 kcal/mol (84 ± 21 kJ/mol), which is close to the values obtained in the course of similar investigations of the Au_n_–biomolecule interactions. One can mention, for example and comparison, the study of Au_5–10_–guanine-cytosine or adenine-uracil to be of about 22–32 kcal/mol or Au_20_–guanine of about 11–16 kcal/mol [14], Au_13_–guanine of about 29 kcal/mol [13], and Au_8,20_–alanine of about 14–22 kcal/mol [11]. It can also be seen in Table 2 that the interaction energy obtained from the reduced models is a reasonable estimation of the values obtained with full models. The predicted interaction energies suggest that the interaction of the Au_n_ cluster with abiraterone at the N-side geometry is preferred over the OH-side geometry. A similar conclusion can be drawn from the predicted interaction energies in the Au_5_–abiraterone acetate conjugate. It is expected that the neutral (zero total electric charge) Au_n_–abiraterone complex becomes a major form to be present in the solution at pH 7.4. As a complementary study, a positively charged complex was considered as well. It contains a protonated abiraterone at the N-nitrogen atom of the pyridine moiety. Such a charged complex is expected to be largely populated in the solution at pH 3.6. Interestingly, in the charged [Au_5_–(NH)abiraterone]^(1+)^ conjugate the geometry where the NH terminal is located nearby the Au_5_ cluster is energetically more favourable than the geometry with the OH terminal in front of Au_5_. However, the interaction energy shows an opposite trend, i.e., the binding energy of Au_5_ in the [Au_5_–(NH)abiraterone)]^(1+)^ conjugate is weaker than in the [Au_5_–(OH)abiraterone]^(1+)^ conjugate.

### 3.3. XRPD Studies

**Figure 6:**
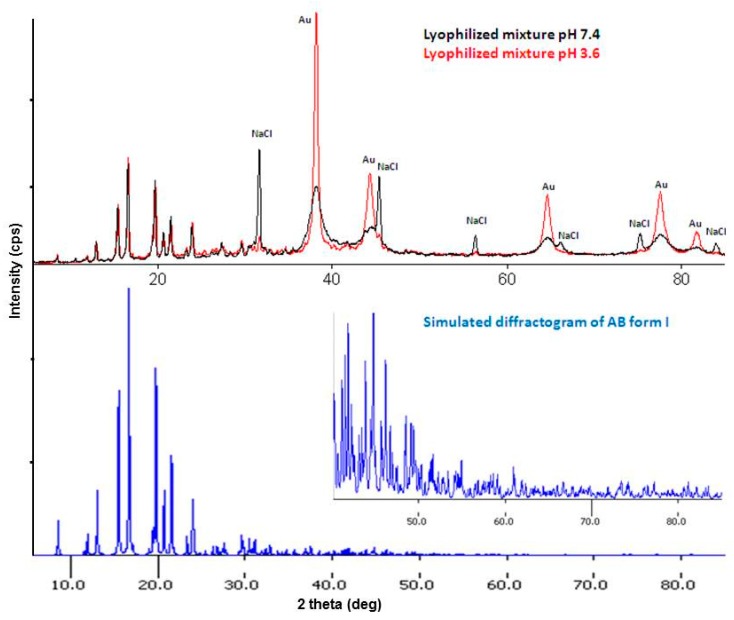
Identification of the crystalline phases in the lyophilized mixtures.

A diffractogram of the lyophilized mixtures obtained in pH 3.6 and 7.4 is characterized by sharp peaks in the range of 5–35° and broad as well as sharp peaks in the range of 35–85° (Figure 6). The identification of the crystalline phases in the mixture diffractogram proved that the sharp peaks in the range of 5–35° belong to abiraterone in the polymorphic form I phase and broad peaks belong to the Au phase (PDF No. 04-0784) [17]. The presence of abiraterone in the polymorphic form I phase in the mixture is confirmed by comparing the mixture diffractogram with the simulated diffractogram of form I [25]. Broad Au peaks indicate small sizes of the crystallites. The FWHM values of the Au(111) peak are 0.5 and 1.4° for the mixtures obtained in pH 3.6 and pH 7.4, respectively. A higher FWHM value for the Au peak in the mixture obtained in pH 7.4 indicates a much smaller size of the Au crystallites. Average sizes of the Au crystallites estimated from the Scherrer formula for this peak are about 19 and 6 nm for the mixtures pH 3.6 and pH 7.4, respectively. Apart from these two phases, the NaCl phase (PDF No. 05-0628) [17] is detected in the mixture obtained in pH 7.4.

Upper window: diffractograms of the mixtures, Au and NaCl peaks are indicated. Below: a simulated diffractogram of abiraterone form I. Insert: a magnification of the low intensity peaks range [25].

**Figure 7:**
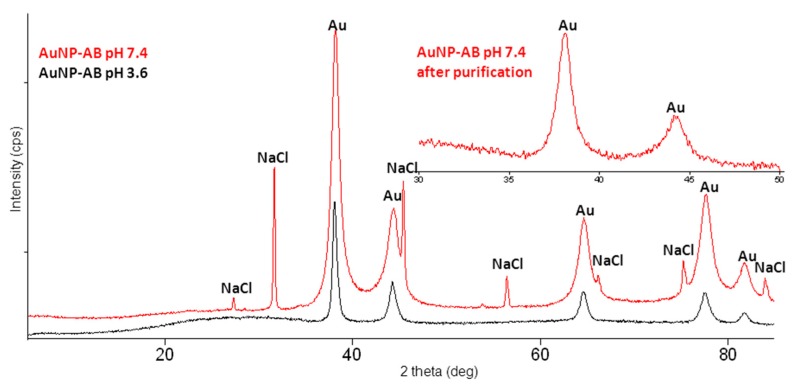
Diffractograms of the AuNPs–AB conjugates obtained in pH 3.6 and 7.4. Insert: a diffractogram of the nanoparticle obtained in pH 7.4 after purification in the range of 30–50°.

Diffractograms of two AuNPs–AB conjugates obtained in pH 3.6 and pH 7.4 are compared in Figure 7. The lack of the AB diffraction peaks suggests its presence in the amorphous content. Broad Au diffraction peaks and sharp peaks of the NaCl phase are best visible in the diffractogram of the nanoparticle obtained in pH 7.4. The FWHM values of the Au(111) peak are 0.5 and 1.0° for the nanoparticles obtained in pH 3.6 and 7.4, respectively. The average sizes of the Au crystallites estimated from the Scherrer formula for this peak are about 17 and 9 nm for the mixtures pH 3.6 and pH 7.4, respectively. An insert in Figure 7 shows that the NaCl phase is removed from the nanoparticle during its purification but the estimated average size of the Au crystallites remains the same.

### 3.4. Raman Spectroscopy Studies

**Figure 8:**
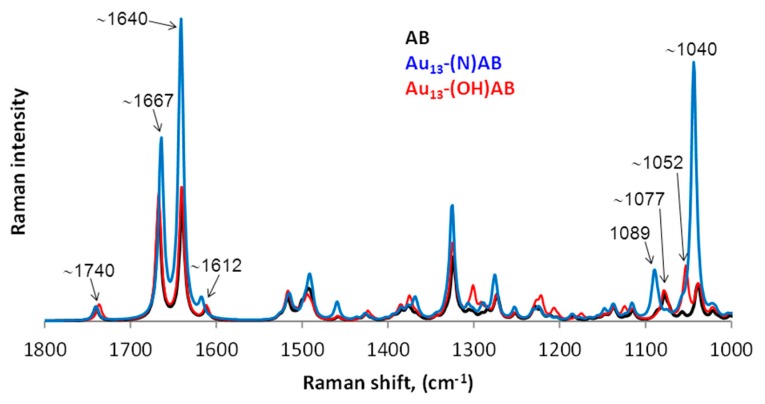
A comparison of the theoretical Raman spectra of AB, Au_13_–(N)AB, and Au_13_–(OH)AB in the range from 1800 to 1000 cm^−1^.

Figure 8 shows a comparison of the theoretical Raman spectra of the AB molecule as well as Au_13_–(N)AB and Au_13_–(OH)AB model nanoconjugates in the range of 1800–1000 cm^−1^. One can see that substantial differences are visible in the ranges of 1660–1580 cm^−1^ and 1100–1000 cm^−1^.

In the theoretical spectra of the AB molecule and both model nanoparticles, the band at 1740 cm^−1^ comes from the stretching vibration of the C=C (B ring) bond. In the spectra of the AB molecule and the Au_13_–(OH)AB model nanoconjugate, the bands at 1667, 1640, and 1612 cm^−1^ originate mainly from the C=C (D ring) bond stretching vibrations and the pyridine ring stretching vibrations. However, in each band the participation of individual vibrations of the C=C (D ring) bond and the pyridine ring is different, which has been summarized in Table 3. For the Au_13_–(N)AB model nanoconjugate, three characteristic bands are observed in the range 1660–1580 cm^−1^: 1664, 1641, and 1617 cm^−1^. The first two bands originate mainly from the C=C (D ring) bond vibrations and the pyridine ring stretching vibrations. These bands are much more intense than their counterparts from the AB and Au_13_–(OH)AB spectra. Just as in the AB and Au_13_–(OH)AB spectra, the third band at 1617 cm^−1^ has small cm^−1^ intensity and originates from the vibration of the entire AB molecule.

In the range of 1100–1000 cm^−1^, two isolated bands at about 1080 and 1040 cm^−1^ are observed in the spectra of AB and the Au_13_–(N)AB model nanoconjugate. The first one comes from the whole AB molecule vibrations in both spectra. The second band comes mainly from the pyridine ring breathing vibrations and the steroid moiety vibrations in the spectrum of the Au_13_–(N)AB model nanoconjugate. This band is much more intense than its counterpart from the AB and Au_13_–(OH)AB spectra. In the spectrum of the Au_13_–(OH)AB model nanoconjugate, three bands at 1039, 1052, and 1076 cm^−1^ are visible. These bands originate from the whole AB molecule vibrations (1076 cm^−1^), steroid moiety vibrations (1052 cm^−1^), and mainly the pyridine ring with the steroid moiety vibrations (1039 cm^−1^). The description of the spectra has been summarized in Table 3.

A significant increase in band intensities in the Au_13_–(N)AB model nanoconjugate spectrum can indicate that the interactions between the Au_13_ cluster and the AB molecule occur via the N atom.

**Figure 9:**
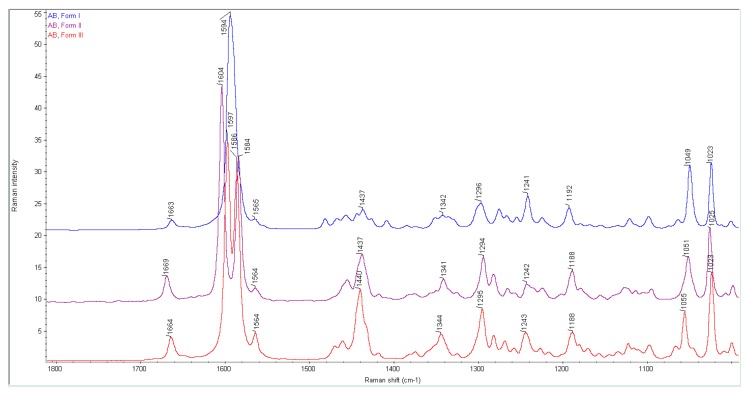
A comparison of the Raman spectra of three AB polymorphs. From the top to the bottom polymorphic spectra I, II, and III are shown.

According to our studies, abiraterone appears in three polymorphic forms: I, II, and III. In Figure 9, the Raman spectra of these forms are collected. The theoretical Raman spectrum is similar to the experimental spectra of forms II and III.

**Figure 10:**
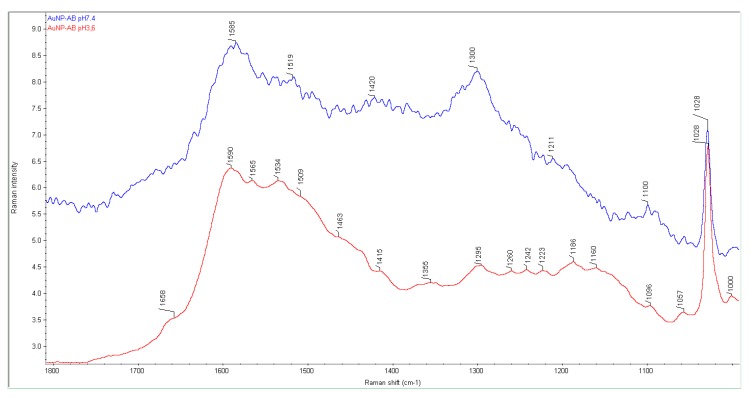
A comparison of the Raman spectra of the nanoparticles obtained in pH 3.6 and 7.4.

Figure 10 shows a comparison of the Raman spectra of the nanoparticles obtained in pH 3.6 and 7.4. It can be seen that the Raman spectrum of the nanoparticle obtained in pH 7.4 is characterized by broad bands at about 1585, 1519, 1420, 1300, 1211, and 1100 cm^−1^ and one characteristic intense narrow band at 1028 cm^−1^. The Raman spectrum of the nanoparticle obtained in pH 3.6 is characterized by broad bands at about 1658, 1590, 1565, 1534, 1509, 1463, 1415, 1355, 1295, 1260, 1242, 1223, 1186, 1160, 1096, and 1057 cm^−1^ and one characteristic intense narrow band at 1028 cm^−1^. This comparison shows that more broadened bands are present in the spectrum of the nanoparticle obtained in pH 7.4. This corresponds well to the smaller Au crystallites estimated by XRPD.

In Table 4, the wavenumbers from the theoretical spectra of AB and Au_13_–(N)AB as well as from three abiraterone forms, in the ranges of 1660–1580 cm^−1^ and at 1100–1000 cm^−1^, have been collected. One can see that the bands in the nanoparticle spectra from the range of 1660–1580 cm^−1^ are shifted towards lower wavenumbers in comparison to the Raman spectra of forms II and III. However, the band at 1028 cm^−1^ visible in the nanoparticle spectra is shifted towards higher wavenumbers in comparison to its counterpart from the spectra of forms I, II, and III (1025 and 1023 cm^−1^, respectively).

The shift into lower wavenumbers in the range of 1660–1580 cm^−1^ and the presence of an intense band at 1028 cm^−1^ in both nanoparticle spectra can suggest that the interactions between the AB molecule and the AuNPs are via the N atom. These studies also prove that the pH changes influence the Au crystalline size in the nanoparticles and bands broadening in the AuNPs–AB spectra.

### 3.5. Thermogravimetric Analysis

The amount of AB on the AuNPs surface was measured using a thermogravimetric analysis (Figure S6). The TGA curve of AB shows a significant decrease in the temperature range from 250 to 600 °C. The mass loss calculated from the AB curve is 95.5%. It means that AB decomposes almost completely in this temperature range. This fact was used in the calculation of the AB quantity in the nanoparticles. The quantity of AB calculated from the TGA curves of AuNPs–AB pH 3.6 and 7.4 is similar and equals 7.8% and 10.2%, respectively.

### 3.6. NMR Studies

Initially, the ^1^H NMR spectrum of the abiraterone acetate nanoparticles was obtained. Signals appeared at approx. 4.43, 5.38, 6.10, 7.33, 7.78, 8.41, and 8.57 ppm. They fit very well with the signals recorded for abiraterone acetate: 4.40 (CH-3), 5.37 (CH-6), 6.10 (CH-16), 7.34 (CH-24), 7.75 (CH-25), 8.40 (CH-23), and 8.56 (CH-21) ppm. This proves that the particles of abiraterone acetate are present in the tested nanoparticle sample. The ^1^H NMR spectra of gold nanoparticles combined with abiraterone were recorded for various synthesis methods. The spectra were obtained with the signals, among others, at approx. 5.29, 6.10, 7.33, 7.75, 8.40, and 8.55 ppm. This matches very well the data obtained for abiraterone measured under similar conditions, where the following signals were obtained: 5.29 (CH-6), 6.10 (CH-16), 7.33 (CH-24), 7.75 (CH-25), 8.55 (CH-23), and 8.56 (CH-21) ppm. This clearly proves that abiraterone is present in the tested samples of the nanoparticles. It is worth noting that in the observed range of the chemical shifts, there is a difference in the chemical shift of the proton of the CH-6 group between abiraterone nanoparticles (5.29 ppm) and abiraterone acetate nanoparticles (5.38 ppm).

**Figure 11:**
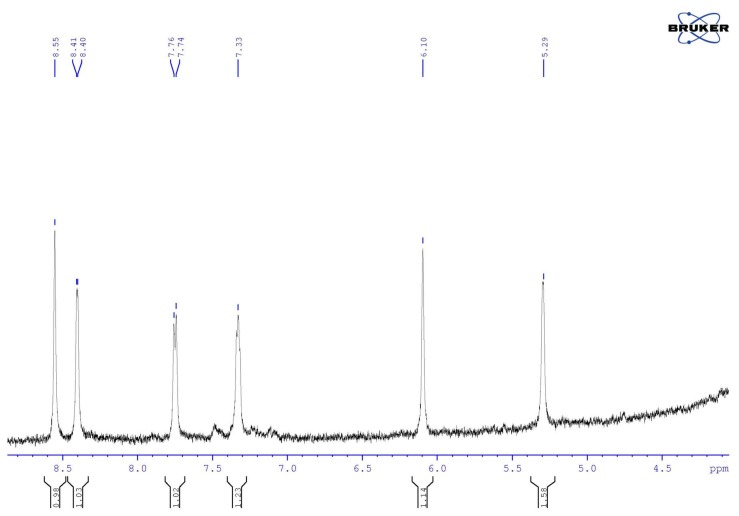
A diagnostic fragment of the NMR spectrum of the abiraterone nanoparticles.

To determine the mode of Au–AB binding, the ^1^H NMR spectra of the nanoparticles (Figure 11) and pure abiraterone measured under similar conditions were analyzed. It was found that the spectra are comparable. The formation of the Au–AB bond does not cause any visible changes in the chemical shifts of the measured protons. Data from the theoretical calculations indicate the nitrogen of the pyridine ring as the Au–AB binding site. Similar chemical shifts observed in the ^1^H NMR spectra of abiraterone nanoparticles and pure abiraterone in the observed range of chemical shifts indicate that the Au–AB binding is weak, hardly affecting the electron density distribution of the neighboring protons. The recorded spectra of the nanoparticles have large intensity signals compared to the abiraterone and abiraterone acetate spectra. This is typical of colloidal solutions, such as nanoparticles, where the solution is heterogeneous as described earlier in the literature [26].

### 3.7. Electrochemistry

**Figure 12:**
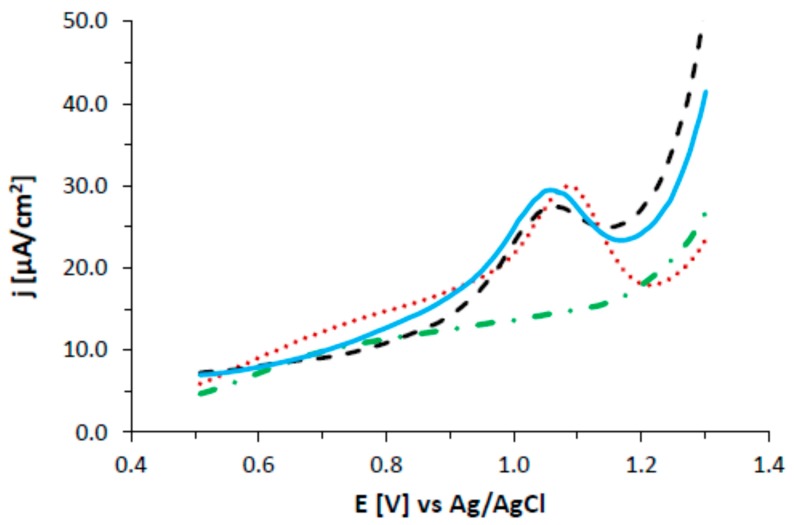
DPV voltammograms (pulse amplitude 50 mV, pulse width 50 ms, potential step 16 mV) recorded using GCE electrodes in deoxygenated DMSO solutions with 0.1 M TBAHFP (green dashed dotted line) background without AB, (red dotted line) with 100 μM AB, and (blue solid line) the AuNPs–AB pH 3.6 conjugate and (dark dashed line) the AuNPs–AB pH 7.4 conjugate transferred to a pure supporting electrolyte.

Electrochemical experiments were carried out using differential pulse voltammetry (DPV) to confirm the formation of gold nanoparticles modified with abiraterone. Electrochemical tests were carried out in a DMSO solution containing 0.1 M TBAHFP (Figure 12). At the beginning, DPV curves were recorded in the DMSO solution containing 0.1 mM abiraterone in the 0.5 V to 1.3 V potential range. A peak at the 1.1 V potential corresponds to the oxidation of abiraterone, because no signals were observed on the DPV curve recorded in the solution without the presence of abiraterone. It is worth nothing that neither the electrochemical behavior of abiraterone itself on a classical electrode nor its electrochemical mechanism have been found in the literature. AB contains cyclohexanol, as well as cholesterol, in its structure, so it was suspected that the electrochemical behavior would be similar. The available literature describes four-electron (4e^−^) oxidation of cholesterol in the cyclohexanol structure, which was manifested by the presence of a peak at the potential of 1.9 V in the acetonitrile solution using a GCE electrode [27,28]. That is why in our research we have attributed the presence of a peak at the potential of 1.1 V to a similar electrochemical process. Then, the electrochemical behavior of two types of AB-modified gold nanoparticles synthesized at pH 3.6 and 7.4 was examined. In both cases, a peak at the potential of 1.05 V was observed on the DPV curves, which corresponds to the oxidation of AB adsorbed on gold nanoparticles. These studies confirm the effective immobilization of AB on gold nanoparticles.

## 4. Conclusions

We have reported a novel method for the successful conjugation of abiraterone and gold nanoparticles, creating a unique nanoconjugate with expected anticancer properties and biomedical applications.

The study comprised as follows:

- synthesis optimization and purification of the AuNPs–AB conjugates that would afford desirable-sized AuNPs,
- theoretical modeling of the interactions of the Au clusters with AB,
- development of the analytical methods for the AB identification in the AuNPs–AB conjugates and the AB quantitation in the supernatants,
- development of analytical methods to study the nanoparticle formation mechanism,
- development of the quantitative methods to estimate the covering of the gold nanoparticles by the AB substance,
- development of an analytical methodology for the physicochemical characterization of the obtained nanoparticle at pH 3.6 and pH 7.4.

Roller-like abiraterone or abiraterone acetate molecules were predicted to form conjugates with small Au clusters at the N-side that would be more stable than those formed at the OH/C=O-sides by about 10 kcal/mol (42 kJ/mol). The binding energy in the conjugates of small Au clusters with abiraterone is predicted to be about 20 kcal/mol (84 kJ/mol) when abiraterone faces Au_n_ at the N-side. The binding energy is smaller, of about 10 kcal/mol (42 kJ/mol), when abiraterone or abiraterone acetate faces Au_n_ at the OH/C=O side.

AB in the gold nanoparticle was identified by electrochemistry and NMR spectroscopy.

The obtained nanoparticles were characterized by the negative zeta potential from −11 to −13 mV, which can suggest their high stability in water and likely low toxicity for normal cells.

The XRPD technique proved the presence of the AuNPs in the lyophilized mixtures as well as in the nanoparticles obtained in pH 3.6 and 7.4. The average size of the Au crystallites estimated from the Scherrer formula was about 9 and 17 nm for the nanoparticles obtained in pH 7.4 and 3.6, respectively. A comparison of the AuNPs–AB precipitates obtained for two different pH values suggests a higher stability of the pH 7.4 conjugate as well as a smaller nanoparticle size as measured by TEM, but the measurements performed by the DLS technique proved that the diameter of the nanoparticle obtained in pH 7.4 is higher than that of the nanoparticle obtained in pH 3.6. In pH 7.4, the nitrogen atom participates in the hydrogen bonds and aggregation may take place: but this, however, requires further research.

The AuNPs–AB conjugates are considered to be candidates for further study and possible application in anticancer therapy.

## Figures and Tables

**Table 1:** Surface Plasmon Resonance (SPR), Hydrodynamic Diameter (DLS), Mean Particle Diameter (TEM), average Au crystallite size (XRPD), and Zeta-Potential Values.

AuNPs–AB	SPR	Diameter (DLS)	Diameter (TEM)	Au Crystallite Size	Zeta Potential, mV
	nm				
pH 3.6	551	229 ± 16	30 (7–65)	17	−11 ± 1
pH 7.4	565	260 ± 35	24 (11–40)	9	−13 ± 1

**Table 2:** Relative stability and binding energy of the Au_n_–AB conjugates and Au_n_–AB reduced models.

Conjugate	Relative Stability ^1)^ kcal/mol (kJ/mol)	Binding Energy ^2)^ kcal/mol (kJ/mol)
**Au_n_–abiraterone**		
Au_5_–(N)abiraterone	0.0	−27.6 (−115.5)
Au_5_–(OH)abiraterone	10.1 (42.1)	−14.5 (−60.6)
Au_13_–(N)abiraterone	0.0	−18.3 (−76.4)
Au_13_–(OH)abiraterone	9.7 (40.6)	−8.6 (−36.1)
**Reduced models**		
Au_13_–(N)pyridine	-	−17.3 (−72.2)
Au_13_–(OH)cyclohexenol	-	−5.3 (−22.2)
Au_20_–(N)pyridine	-	−18.7 (−77.0)
Au_20_–(OH)cyclohexenol	-	−9.2 (−38.5)
**Au_n_–abiraterone acetate**		
Au_5_–(N)abiraterone acetate	0.0	−25.9 (−108.3)
Au_5_–(O=C)abiraterone acetate	10.5 (44.1)	−15.9 (−66.6)
**Charged models**		
[Au_5_–(NH)abiraterone]^1+^	0.0	−7.8 (−32.5)
[Au_5_–(OH)abiraterone]^1+^	11.9 (49.7)	−12.8 (−53.5)

^1)^ The relative stability is based on the comparison of the calculated free energies of the conjugate. ^2)^ The binding energy is calculated for the conjugates using the counterpoise correction for the intermolecular interactions.

**Table 3:** A description of characteristic bands from the theoretical spectra of AB, Au_13_–(N)AB, and Au_13_–(OH)AB.

AB		Au_13_–(N)AB ^1)^		Au_13_–(OH)AB	
Wavenumbers, (cm^−1^)					
1740	C=C (B) ^2)^	1740	C=C (B)	1737	C=C (B)
1667	mainly C=C (D) ^3)^ + pyridine	1665	mainly C=C (D) + pyridine	1667	mainly C=C (D) + pyridine
1640	pyridine	1641	mainly pyridine + C=C (D)	1640	pyridine
1612	mainly pyridine + C=C (D)	1617		1612	mainly pyridine + C=C (D)
1078	whole AB molecule	1089	entire AB molecule	1076	entire AB molecule
--	--	--	--	1052	steroid moiety with OH without pyridine
1040	whole AB molecule	1043	mainly pyridine + steroid moiety with OH	1039	mainly pyridine + steroid moiety

^1)^ wavenumbers for the Au_5_–(N)AB model are the same. ^2), 3)^ description of the AB rings from Figure 1.

**Table 4:** A collection of the wavenumbers from the theoretical and experimental Raman spectra of AB and the nanoparticles.

Theoretical		Experimental				
AB	Au_13_–(N)AB	Form I	Form II	Form III	AuNPs–(N)AB pH 3.6	AuNPs–(N)AB pH 7.4
Wavenumbers, (cm^−1^)						
1740	1740	1663	1669	1664	1658	--
1667	1664	1594	1604	1597	1590	1585
1640	1641	--	1584	1586	1565	--
1612	1617	1565	1564	1564	1534	1519
1078	1089	1049	1051	1055	1057	--
1040	1043	1023	1025	1023	1028	1028

## Supplementary Materials

The following are available online at http://www.mdpi.com/2079-4991/8/9/641/s1.

## Abbreviations

Au-NPs	gold nanoparticles
AB	abiraterone
DLS	dynamic light scattering
XRPD	X-ray powder diffraction
TEM	transmission electron microscopy
MeCN	acetonitrile
*n*-BuOH	*n*-butanol
DMSO	dimethyl sulfoxide
DMF	dimethylformamide
THF	tetrahydrofuran
