# Supplementary material for: Design and Molecular Modeling of Abiraterone-Functionalized Gold Nanoparticles
Source: Nanomaterials (Basel). 2018 Aug 22;8(9):641. doi: 10.3390/nano8090641 (PMC6164775; doi:10.3390/nano8090641)
Supplement: Supplementary file 1 [file nanomaterials-08-00641-s001.pdf]

Supplementary Material

Design and Molecular Modeling of Abiraterone-Functionalized Gold Nanoparticles

Elżbieta U. Stolarczyk[1,*], Marta Łaszcz[1], Andrzej Leś[1, 2], Marek Kubiszewski[1], Krzysztof Kuziak[1], Katarzyna Sidoryk[3], Krzysztof Stolarczyk[2]

[1]*Pharmaceutical Research Institute, R&D Analytical Department, 8 Rydygiera Str., 01-793 Warsaw, Poland*

[2]*University of Warsaw, Faculty of Chemistry, 1 Pasteura Str., 02-093 Warsaw, Poland*

[3]*Pharmaceutical Research Institute, Chemistry Department, 8 Rydygiera Str., 01-793 Warsaw, Poland*

Author to whom correspondence should be addressed:

[*] Pharmaceutical Research Institute, R&D Analytical Department, 8 Rydygiera Str., 01-793 Warsaw, Poland, tel. +48 22 456 3992

e-mail: e.stolarczyk@ifarm.eu

1. Elements of the validation of the UV-Vis method for the determination of AB residual in the supernatant

Abiraterone UV-Vis spectrum in n-BuOH shows λ_{max} at 255 nm.

Linearity: The calibration curve was obtained in the concentration range from 4 μg/mL – 50 μg/mL. For each concentration three repetitions were performed for the average result. Three

replicate injections were made for each concentration and the average result was reported. The response of abiraterone was found to be linear in the investigated concentration range and the linear regression equation was y = 0.028x – 0.0069 with the correlation coefficient equal 0.9997 (Table S1, Figure S1).

Table S1. Results of the method linearity test.

**Linearity of the abiraterone determination**						
**Solution No.**	**Concentration, µg/mL**	**Abs**	**Mean Absorbance (Abs)**	**SD Abs**	**RSD Abs %**	**Abs /Conc**
1	**4.0**	0.114	0.114	0.0000	0.00	0.029
		0.114				0.029
		0.114				0.029
2	**5.0**	0.138	0.137	0.0010	0.73	0.028
		0.136				0.027
		0.137				0.027
3	**10.1**	0.269	0.270	0.0006	0.22	0.027
		0.270				0.027
		0.270				0.027
4	**20.1**	0.551	0.551	0.0000	0.00	0.027
		0.551				0.027
		0.551				0.027
5	**30.2**	0.834	0.834	0.0006	0.07	0.028
		0.833				0.028
		0.834				0.028
6	**40.2**	1.112	1.110	0.0021	0.19	0.028
		1.109				0.028
		1.108				0.028
7	**50.3**	1.414	1.414	0.0000	0.00	0.028
		1.414				0.028
		1.414				0.028
					**Mean**	0.028
					**SD**	0.001
					**RSD**	1.991

Table: Analysis of the linear regression y = 0.0281x – 0.0069			
R^2		0.9997	
Number of data		7.00	
Standard deviation SD_{xy}		0.009	
Coefficient a (slope):			
		a	0.028
		Standard deviation SDa	0.0002
		$t_a=\|a\|/SD_a$	135.16
		t_{cr} (a=0.05: f=n-2)	2.57
**Conclusion**	$t_a \gg t_{cr}$		
	**Coefficient *a* is significant, the method is sensitive**		
Coefficient b (y-intercept):			
		b	-0.007
		Standard deviation SD_b	0.006
		$t_b=\|b\|/SD_b$	1.183
		t_{cr}(a=0.05: f=n-2)	2.57
**Conclusion**	$t_b < t_{cr}$		
	**Coefficient *b* is not significant, the method has no systematic errors**		
Coefficient r:			
		r	0.9999
		r sq	0.9997
		tr=r/√(1-r^2)×√(n-2)	135.16
		t_{cr}(a=0.05: f=n-2)	2.57
**Conclusion**	tr $\gg t_{cr}$		
	**Coefficient *r* is significant, the method is linear**		

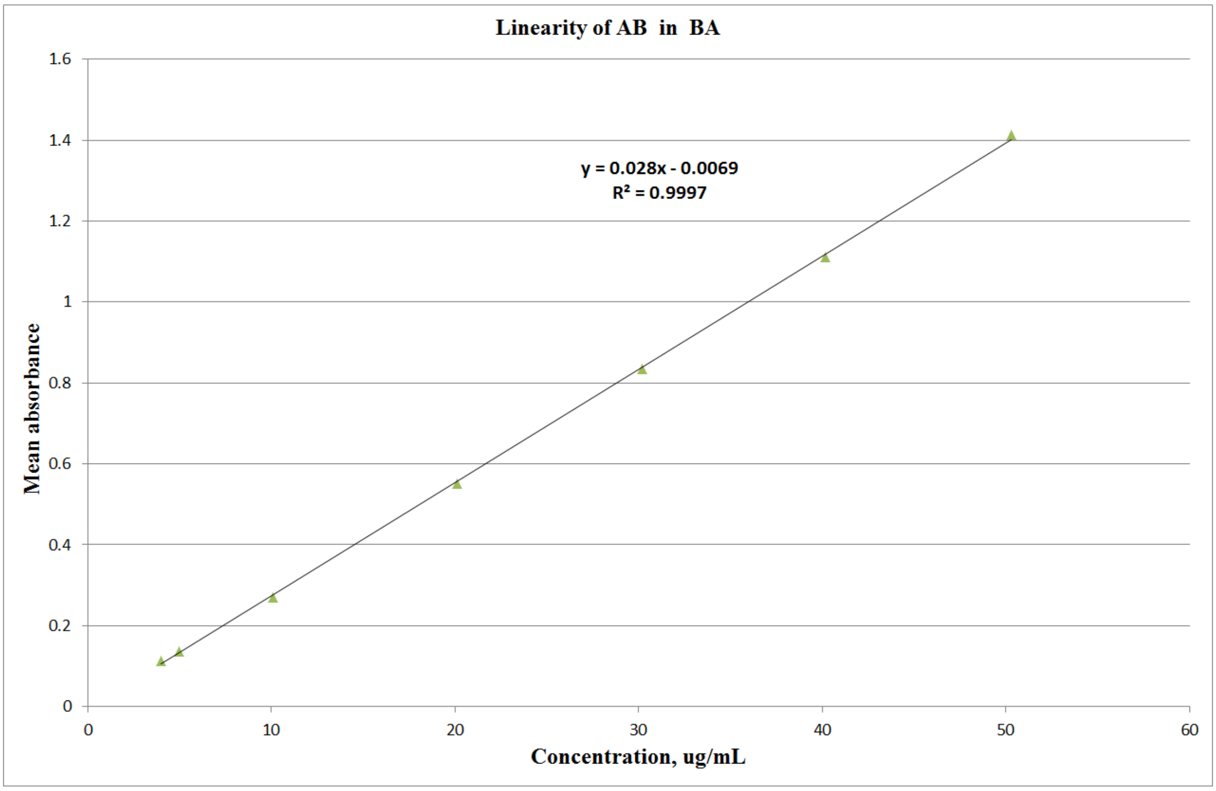

Figure S1. Linearity of the abiraterone determination.

Precision: precision was evaluated by measuring the response of six replicate solutions with the analytes at 50 µg/mL (Table S2).

Table S2. Results of the precision test for abiraterone.

**Sample No.**	**AB**	
	**Abs**	**λ_{max}**
1	1.409	254.669
2	1.407	254.369
3	1.410	254.888
4	1.408	254.755
5	1.408	254.725
6	1.408	254.415
**Mean**	1.408	254.637
**SD**	0.001	0.20337
**RSD %**	0.073	0.079

The limit of detection (LOD): LOD is defined as the lowest concentration of an analyte that an analytical process can reliably differentiate from the back-ground levels. Solutions of different lowering concentrations of AB were analysed.

The limit of quantification (LOQ): LOQ is defined as the lowest concentration of the standard that can be measured with an acceptable precision and linearity. Solutions of different lowering concentrations of AB were analysed. The precision of the LOQ level was established by measuring the response of six replicate measurements of the LOQ solution for AB (Table S3) .

Detection and quantification limits were found to be 3 μg/mL and 4 μg /mL, respectively.

Table S3. Results for the examined LOQ solutions.

**Sample No.**	**AB**	
	**Abs**	λ_{max}
1	0.114	254.594
2	0.114	254.167
3	0.114	254.618
4	0.114	254.126
5	0.114	254.448
6	0.114	254.680
**Mean**	0.114	254.4388
**SD**	0	0.239241
**RSD %**	0	0.094027

2. Theoretical studies

The selected geometry parameters are collected in Table S4. An interesting feature of the small Au_n clusters is their irregular (non-symmetrical) structure, except for Au_{20} which is close to a tetrahedral structure.

Table S4. Jmol structures of AB acetate and its complexes with Au clusters.

AB
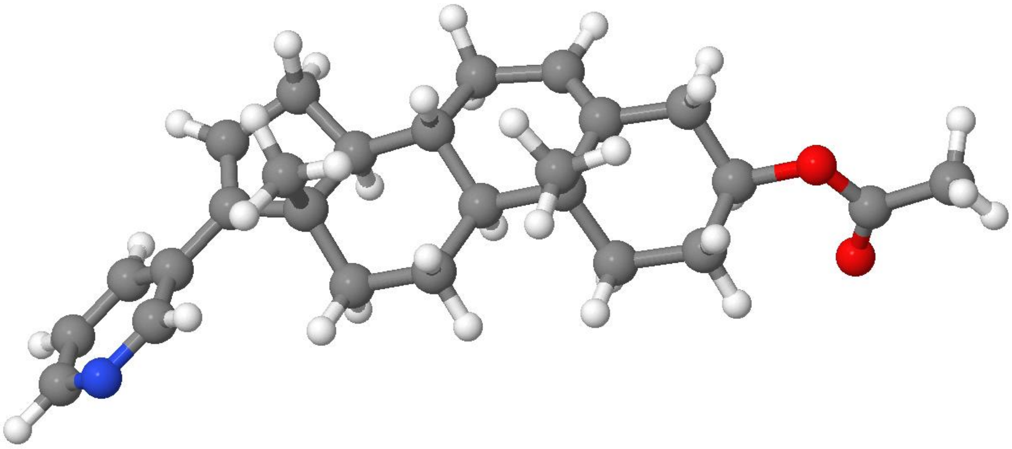
Au_5
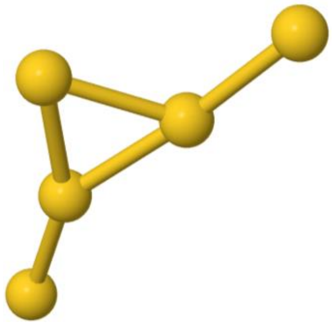
Au_8
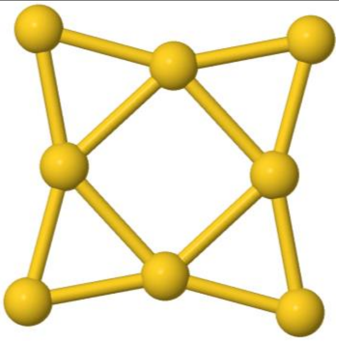
Au_{13}

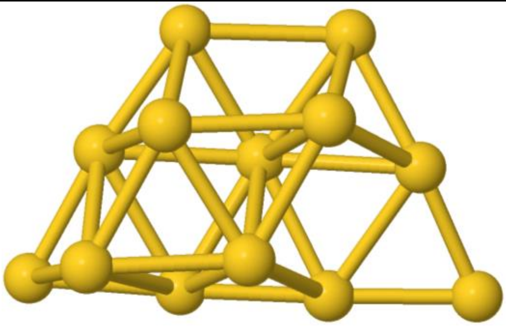

Au_{20}

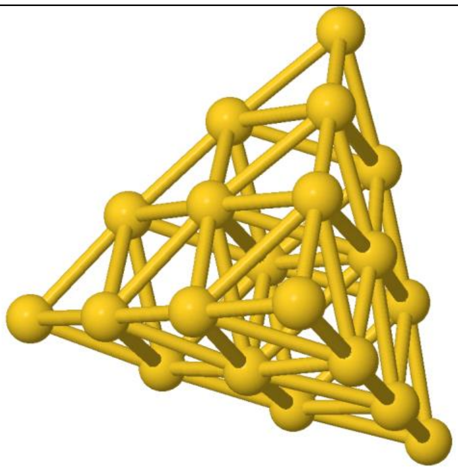

AuNPs–(N)AB acetate for Au_5

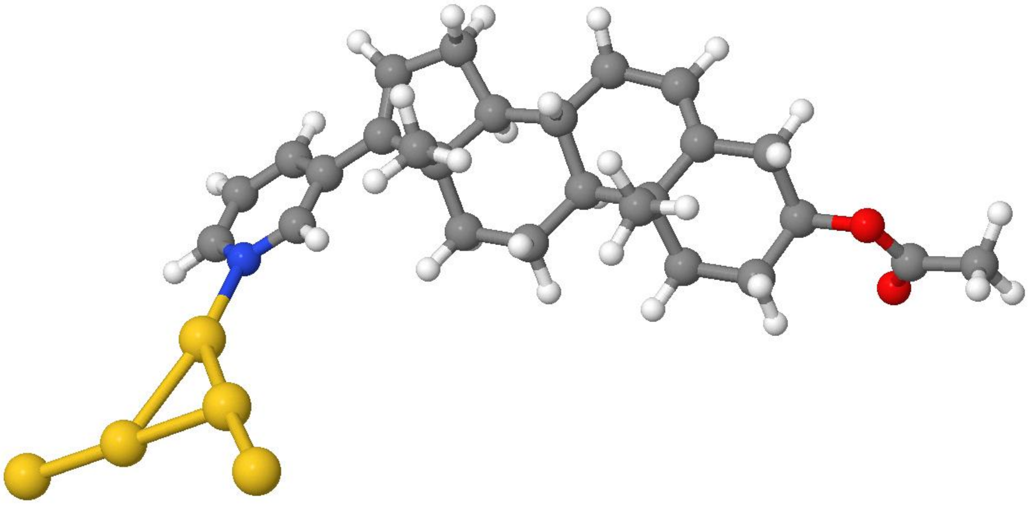

AuNPs–(O)AB acetate

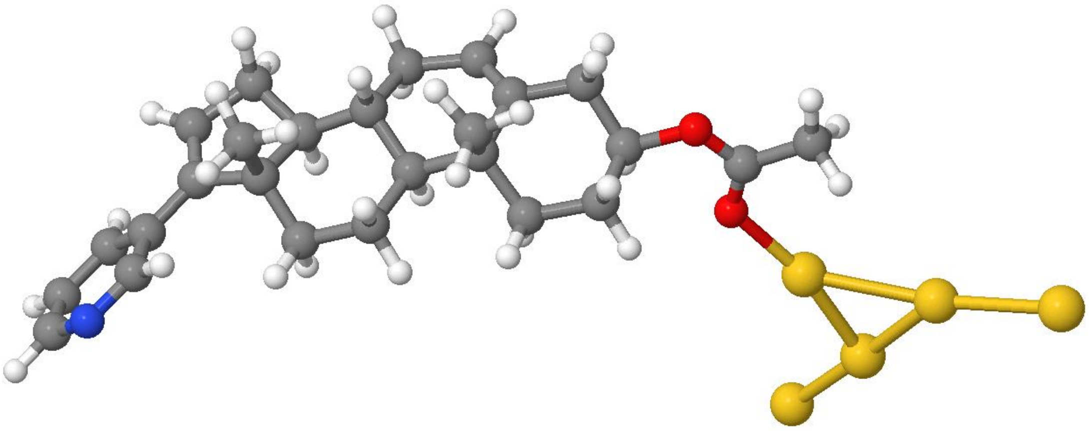

Jmol structures of AB and its complexes with Au clusters

AB

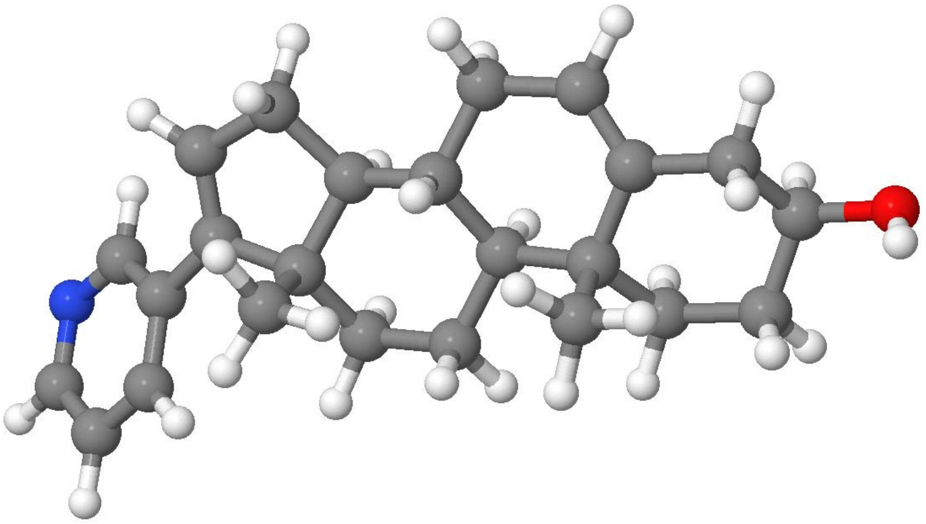

AuNPs–(N)AB for Au_5

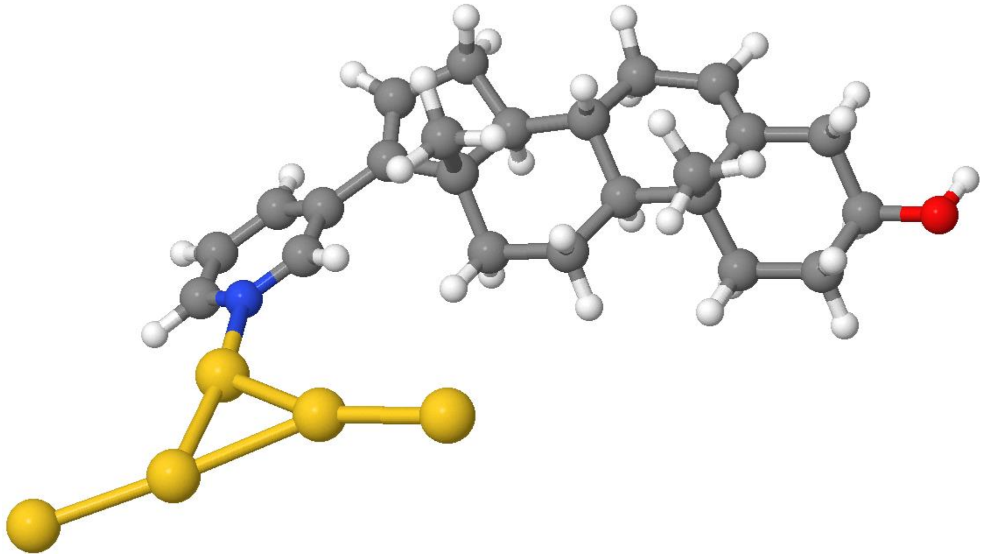

AuNPs–(N)AB for Au_{13}
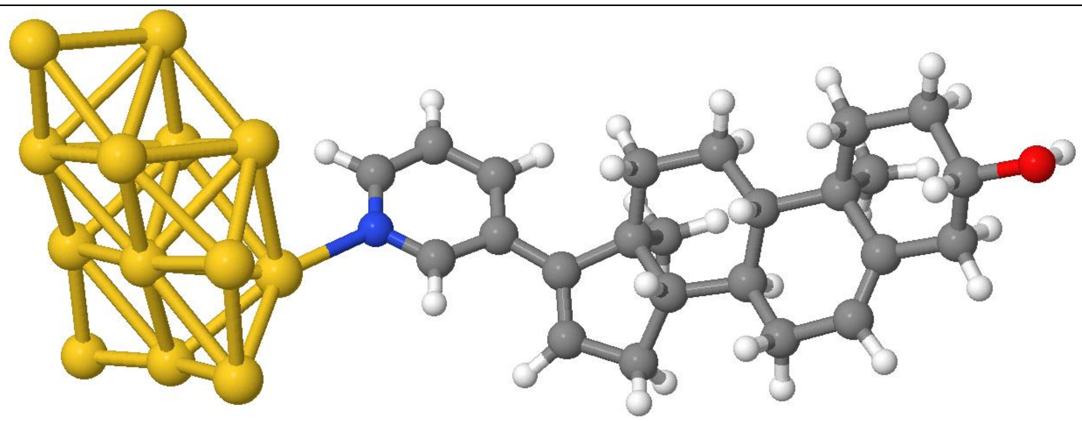
AuNPs–(OH)AB for Au_{13}
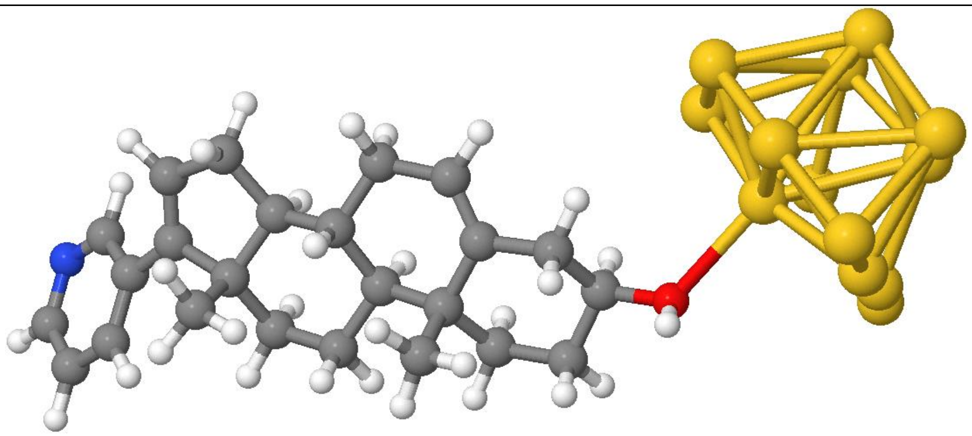

Footnote to Table S4. Distances between neighbouring atoms, in nm, in the Au_n clusters predicted with the present theoretical calculations.

Au_n	Distances
Au_5	26.2-27.9
Au_8	27.0-28.7
Au_{13}	27.0-30.2
Au_{20}	27.3-32.1

Table S5. Intermolecular interaction energies of the Au_n–abiraterone conjugates calculated with the use of the counterpoise correction. The A symbol corresponds to Au_n, while the B symbol to the abiraterone molecule (neutral/protonated/acetated) or to its reduced models.

Conjugate	E(AB)	E(A)	E(B)	Eint [kcal/mol]	Eint [kJ/mol]
**Au_n–abiraterone**					
Au_5–(N)abiraterone	-1740.655750<br>-1740.229974[1)]	-677.410718	-1063.201028	-27.6	-115.5
Au_5–(OH)abiraterone	-1740.638319<br>-1740.213932[1)]	677.412515	-1063.202718	-14.5	-60.6
Au_{13}–(N)abiraterone	-2824.694197<br>-2824.301587[1)]	-1761.462732	-1063.202378	-18.25	-76.37
Au_{13}–(OH)abiraterone	-2824.684597	-1761.466530	-1063.204306	-8.6	-36.1

	-2824.286120[1)]				
**Reduced models**					
Au_{13}–(N)pyridine	-2009.783477	-1761.463578	-248.292406	-17.3	-72.2
Au_{13}–(OH)cyclohexenol	-2071.354302	-1761.467800	-309.878060	-5.3	-22.2
Au_{20}–(N)pyridine[2)]	-2958.376778	-2710.108047	-248.239413	-18.4	-77.0
Au_{20}–(OH)cyclohexenol[2)]	-3019.945856	-2710.107951	-309.823240	-9.2	-38.5
**Charged models**					
[Au_5–(NH)abiraterone]$^{(+)}$	-1741.036293<br>-1740.600857[1)]	-677.436509	-1063.587416	-7.8	-32.5
[Au_5–(OH)abiraterone]$^{(+)}$	-1741.022991<br>-1740.581909[1)]	-677.411926	-1063.590688	-12.8	-53.5
**Au_n–abiraterone acetate**					
Au_5–(N)abiraterone acetate	-1893.322625<br>-1892.848745[1)]	-677.412495	-1215.868885	-25.9	-108.3
Au_5–(O=C)abiraterone acetate	-1893.306051<br>-1892.848745[1)]	-677.412122	-1215.868551	-15.92	-66.6

1) The sum of electronic and thermal free energy

2) Calculations using smaller Gaussian basis set D95V [20]

3. Lyophilized mixtures

3.1. Abiraterone acetate

3.1.1. XRPD studies

The diffractogram of the lyophilized mixture is characterised by sharp peaks in the range of 5 – 35 ^{o} and broad peaks in the range of 35 – 85 ^{o} (Figure S2). Identification of the crystalline phases in the mixture diffractogram proved that the sharp peaks belong to the AB acetate phase and the broad peaks belong to the Au phase (PDF no 04-0784) [14]. The presence of AB acetate in the mixture is confirmed by the comparison of the mixture diffractogram with a simulated diffractogram of AB acetate [19]. Broad Au peaks indicate small sizes of the crystallites. The FWHM value for the Au (111) peak at 38.2 ^{o} is 1.4 ^{o}. The average size of the Au crystallites estimated from the Scherrer formula for this peak is about 6 nm.

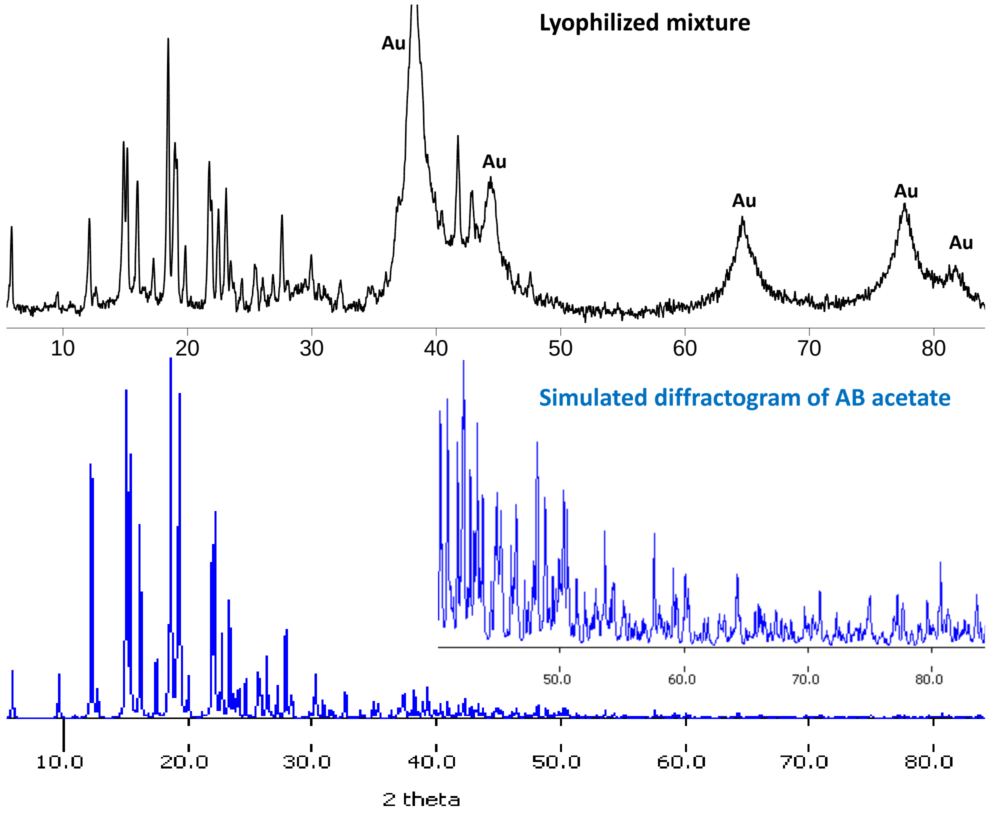

Figure S2. Identification of the crystalline phases in the lyophilized mixture.

Upper window: a mixture diffractogram, Au peaks are indicated.

Below: the simulated diffractogram of AB acetate, an insert: a magnification of low intensity peaks range.

4. NP-based system

4.1. AuNPs-AB acetate

4.1.1. XRPD

Figure S3 shows a diffractogram of the AuNPs–AB acetate conjugate. The lack of the AB acetate diffraction peaks suggests its presence in the amorphous content. Broad Au diffraction peaks are best visible. The FWHM value of the Au (111) peak is 0.6 ^{o}. An average size of the Au crystallites estimated from the Scherrer formula for this peak is about 14 nm.

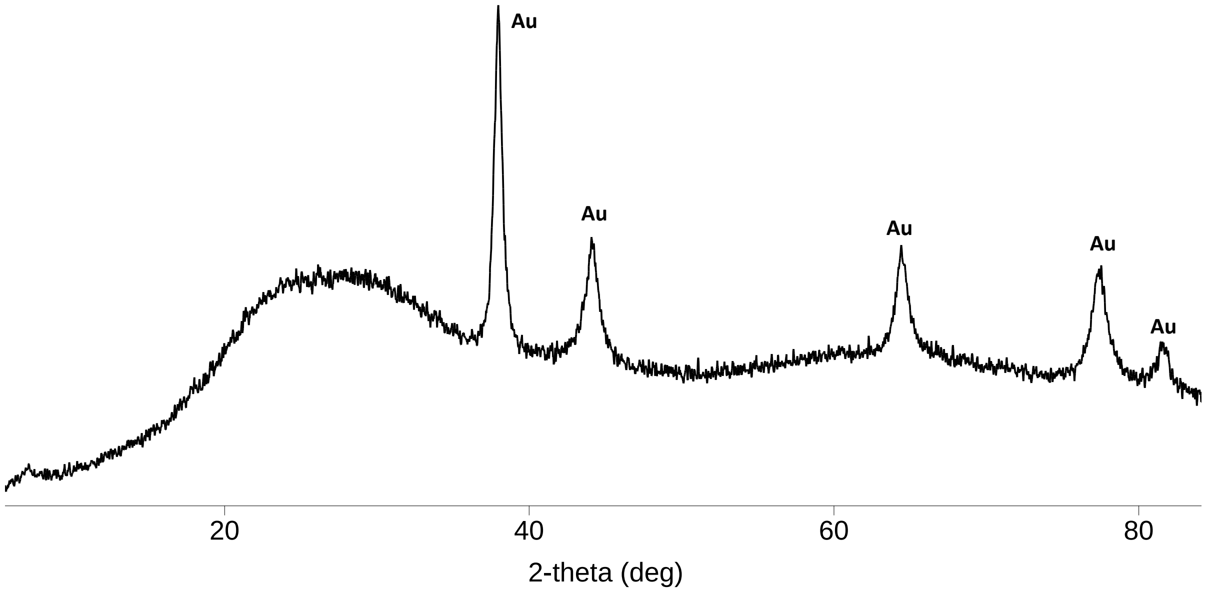

Figure S3. A diffractogram of the AuNPs–AB acetate conjugate.

4.1.2. Raman

Figure S4 shows a comparison of the theoretical Raman spectra of AB acetate as well as the Au_5–(N)AB acetate and Au_5–(O)AB acetate conjugate in the range from 1800 to 1000 cm^{-1}. This comparison demonstrates that the same band is observed in the three spectra at

about 1740 cm^{-1}, coming from the C=C (B) stretching vibrations. Substantial differences occur in the ranges of 1700 – 1600 cm^{-1} and in 1100 – 1000 cm^{-1}.

In the spectrum of Au_5–(O)AB acetate a characteristic band at 1692 cm^{-1}, originating from the C=O stretching vibrations, is observed. In all studied spectra the band at about 1660 cm^{-1} comes from the collaborative stretching vibrations of mainly C=C (D) bond and the pyridine ring. The band at about 1640 cm^{-1} comes from the stretching vibrations of the pyridine ring in the AB acetate spectrum, but in the nanoparticle spectra this band comes from the collaborative vibrations of the pyridine ring and the C=C (D) bond.

In the range of 1100–1000 cm^{-1}a very intensive band at 1045 cm^{-1} is observed , in the spectrum of Au_5–(N)AB acetate, originating from the pyridine ring vibrations. In the spectra of AB acetate and Au_5–(O)AB acetate the band at 1040 cm^{-1} comes from the AB acetate molecule vibrations. The description of the spectra is summarised in Table S6.

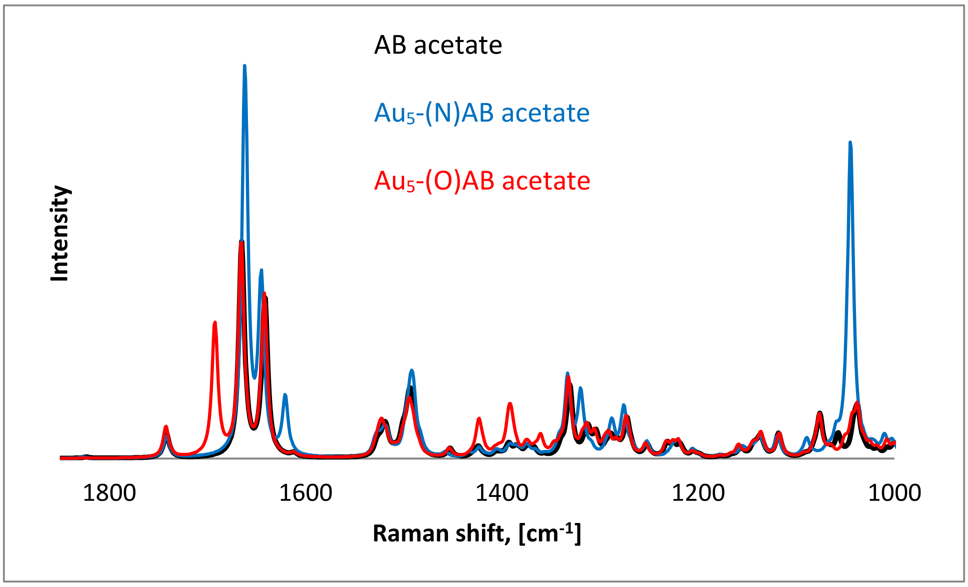

Figure S4. A comparison of the theoretical Raman spectra of AB acetate and the nanoparticles of Au_5–(N)AB acetate and Au_5–(O)AB acetate in the range from 1800 to 1000 cm^{-1}.

Table S6. A description of the characteristic bands in the theoretical spectra of AB acetate and the nanoparticles of Au_5–(N)AB acetate and Au_5–(O)AB acetate.

**AB**		**Au_5–(N)AB acetate**		**Au_5–(O)AB acetate**	
Raman shifts, (cm^{-1})					
1824	C=O	--	--	--	--
1742	C=C (B)	1742	C=C (B)	1742	C=C (B)
--	--	--	--	1692	C=O
1665	mainly C=C (D) + pyridine	1662	mainly C=C (D) + pyridine	1666	mainly C=C (D) + pyridine
1641	Pyridine ring	1645	mainly pyridine + C=C (D)	1642	mainly pyridine + C=C (D)
--	--	1621		--	--
1076	whole AB acetate molecule	1069	steroid moiety with acetate without the pyridine ring	1076	whole AB acetate molecule
1036	whole AB acetate molecule	1045	pyridine	1038	whole AB acetate molecule

Experimental Raman spectra of AB acetate and the AuNPs–AB acetate conjugate are compared in Figure S5. The experimental spectrum of AB acetate is similar to the calculated one.

The experimental spectrum of the AuNPs–AB acetate conjugate is characterised by very broad bands at about 1579, 1446, 1378, 1310, 1264, and 1142 cm^{-1}. One characteristic intense narrow band at 1028 cm^{-1} is observed. This band is shifted 4 cm^{-1} into higher wavenumbers in comparison with its counterpart in the experimental AB acetate spectrum which indicates an interaction between the AuNPs and AB acetate. The direction of this shift agrees well with theoretical predictions, but it is difficult to conclude on the manner of the interaction because the experimental spectrum of AuNPs–AB acetate is characterised by very broad bands in the range of 1800–1500 cm^{-1}.

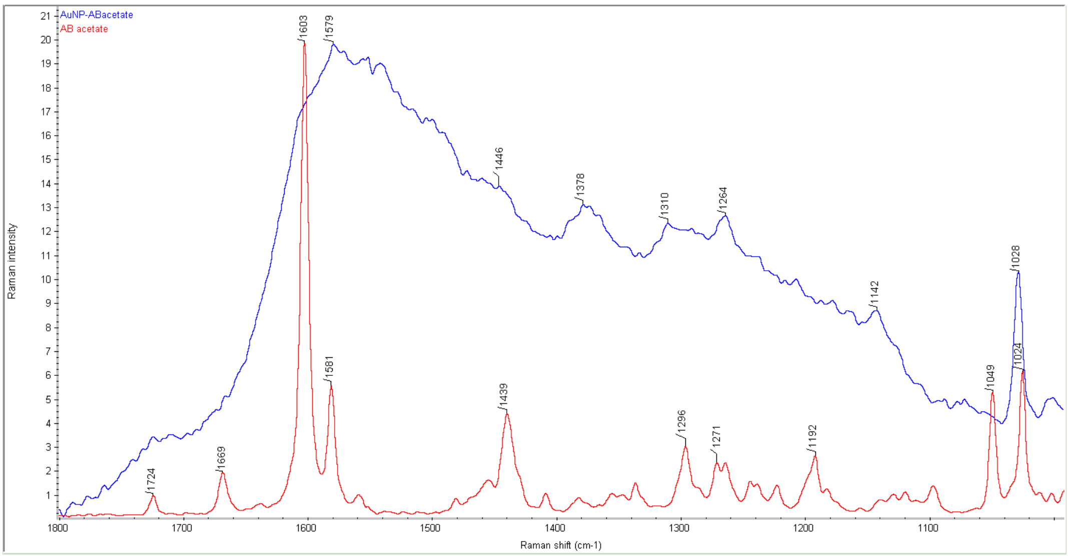

Figure S5. A comparison of the experimental Raman spectra of AB acetate (red spectrum) and AuNPs–AB acetate (blue spectrum).

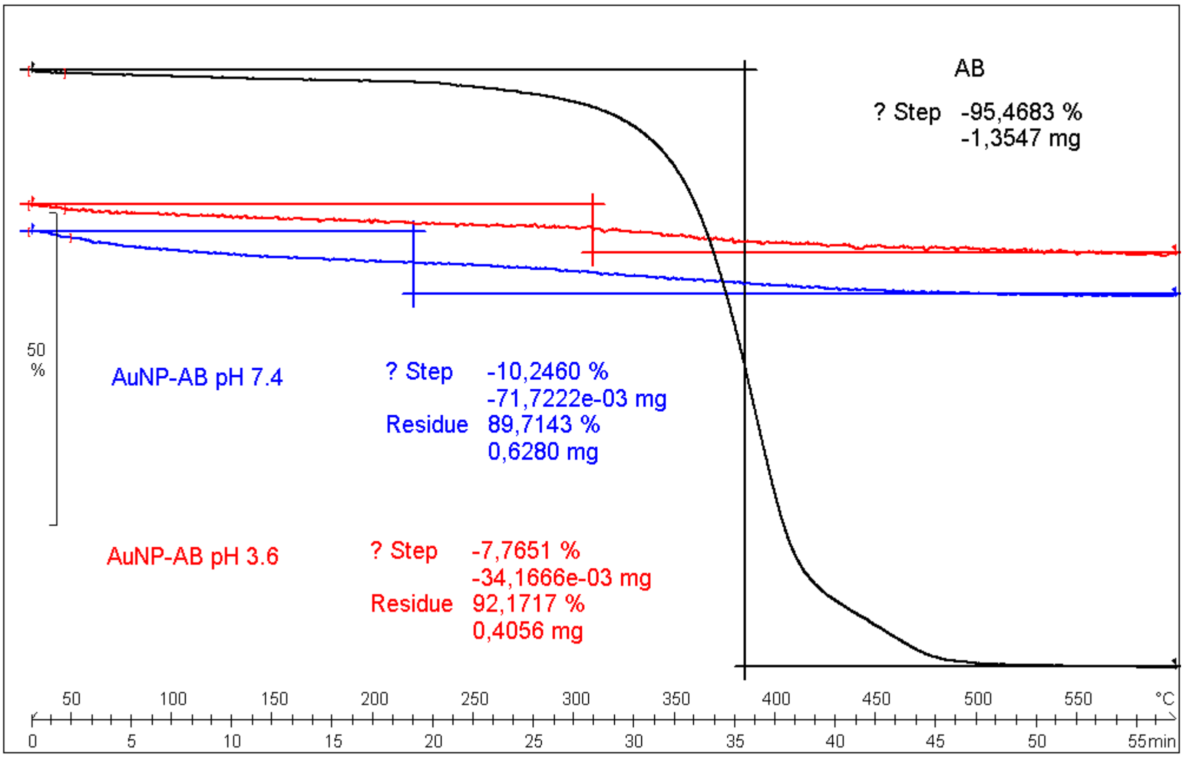

Figure S6. TGA curves of AB and the nanoparticles.
